# Visual measurement of the evaporation process of a sessile droplet by dual-channel simultaneous phase-shifting interferometry

**DOI:** 10.1038/srep12053

**Published:** 2015-07-16

**Authors:** Peng Sun, Liyun Zhong, Chunshu Luo, Wenhu Niu, Xiaoxu Lu

**Affiliations:** 1 Guangdong Provincial Key Laboratory of Nanophotonic Functional Materials and Devices, South China Normal University, Guangzhou 510006, China

## Abstract

To perform the visual measurement of the evaporation process of a sessile droplet, a dual-channel simultaneous phase-shifting interferometry (DCSPSI) method is proposed. Based on polarization components to simultaneously generate a pair of orthogonal interferograms with the phase shifts of π/2, the real-time phase of a dynamic process can be retrieved with two-step phase-shifting algorithm. Using this proposed DCSPSI system, the transient mass (TM) of the evaporation process of a sessile droplet with different initial mass were presented through measuring the real-time 3D shape of a droplet. Moreover, the mass flux density (MFD) of the evaporating droplet and its regional distribution were also calculated and analyzed. The experimental results show that the proposed DCSPSI will supply a visual, accurate, noncontact, nondestructive, global tool for the real-time multi-parameter measurement of the droplet evaporation.

Study on the droplet evaporation is very meaningful, such as spraying of pesticides^1^, the engine fuel injection^2^, biological tissue culture^3^, deposition of DNA/RNA micro-array^4^,^5^, thin film coatings^6^,^7^ as well as the natural phenomena such as weather estimates of rain, dew, snow^8^, etc. Though a lot of methods have been employed to study the droplet evaporation, such as tensiometer^9^, quartz crystal microbalance^10^,^11^, and the optical method such as the photography was also used when measuring the contact radius and the contact angle of droplet^11^, it is still difficult to perform the real-time 3D shape measurement of the evaporating droplet^9^,^12^. Numerous theories^13^ regarding the droplet evaporation were based on the idealized segment model, in which the droplet was considered very small in size and deposited on the surface of a isotropic substrate, and the surface tension of droplet was more than the gravity. After getting the 2D image of droplet by the optical method, 3D shape of the evaporating droplet was determined by mathematical derivation and calculation. To date, there is no comprehensive understanding about the evaporation process of a droplet, especially in the case of a complex environment or some compound droplets, and the precise mechanism of the droplet evaporation still needed to be further studied. Clearly, the real-time 3D shape measurement of the droplet is an important research content of the droplet evaporation.

Phase-shifting interferometry (PSI)^14^, which is a highly accurate, noncontact, global and fast interferometry, has been extensively used in various metrological fields, such as optical surface analysis, optical microscopy and digital holography. Conventional temporal phase-shifting interferometry (TPSI) cannot be implemented in the phase measurement of a dynamic process, because three or more phase-shifting interferograms captured at different time are required. In order to perform the phase retrieval of a moving object or dynamic process, Fourier transform (FT) method and spatial phase-shifting interferometry (SPSI)^15^,^16^,^17^,^18^,^19^,^20^,^21^,^22^,^23^ have been introduced. FT method^15^ can conveniently retrieve the measured phase from only one interferogram, but its spatial carrier frequency will affect the recording of object’s high-frequency information, moreover, the accuracy of phase retrieval is closely associated with the filter window and the uniformity of interferogram. In SPSI, by using some polarization components and beam splitters, three or four phase-shifting interferograms can be captured simultaneously by three or four CCD cameras or three or four areas on a single CCD. To date, three methods were employed to record phase-shifting interferograms in SPSI: (1) multi-frame interferograms with different phase shifts are simultaneously captured on different areas of a single CCD^17^,^20^,^21^; (2) three or four phase-shifting interferograms are simultaneously captured by three or four CCDs^16^,^19^,^23^, respectively; (3) multi-group four-step phase-shifting interferograms are generated by a pixelated phase-mask, in which each pixel has a unique phase-shifts and four adjacent pixels are arranged into a “unit cell” to generate a group of four-step phase-shifting interferograms. When unit cell is repeated continuously over the entire CCD array, multi-group four-step phase-shifting interferograms are generated by a single CCD^22^. Since multi-frame phase-shifting interferograms were captured simultaneously, SPSI can effectively restrain the noise, it should be a better candidate suitable for the phase measurement of a dynamic process.

Recently, many two-step phase retrieval algorithms from two-frame phase-shifting interferograms with unknown phase shifts have been reported^24^,^25^,^26^,^27^,^28^. Kreis *et al.* first proposed a fundamental two-step phase demodulation algorithm by applying the spatial Fourier transform and the filtering processing^24^. Vargas *et al.* proposed a regularization optical flow algorithm^25^, in which the phase shifts between two-frame interferograms with arbitrary value can be obtained. However, since the parameters of the interference fringe direction were determined by the iterative calculation, this phase retrieval algorithm was time-consuming. To address this, Vargas *et al.* introduced Gram–Schmidt (GS) orthonormalization algorithm with high accuracy and rapid calculation speed, in which two orthonormal vectors coming from two corresponding interferograms were constructed for the phase demodulation^26^. Moreover, based on the ratio of the extreme values of interferograms (EVI) to estimate the phase shifts, we proposed a useful phase reconstruction algorithm. In the case that the fringe number of interferogram was less than one and all other two-step algorithms were unsuccessful, the phase still can be retrieved with this EVI algorithm^27^. Recently, from three interferograms with unknown phase shifts, we introduced a novel phase retrieval algorithm, in which though two-step phase-shifting algorithms were employed^28^, but the calculation speed and the accuracy with the proposed algorithm were greatly better than that from only two phase-shifting interferograms.

In this study, in order to perform the phase retrieval of a dynamic process, we proposed a dual-channel simultaneous phase-shifting interferometry (DCSPSI). Using this DCSPSI system, we have implemented the real-time 3D shape measurement of the evaporation process of a droplet. Following, we first introduced the principle of the DCSPSI method, and then presented the experimental results of the evaporation process of a sessile droplet with different initial mass.

## Methods

**Figure 1:**
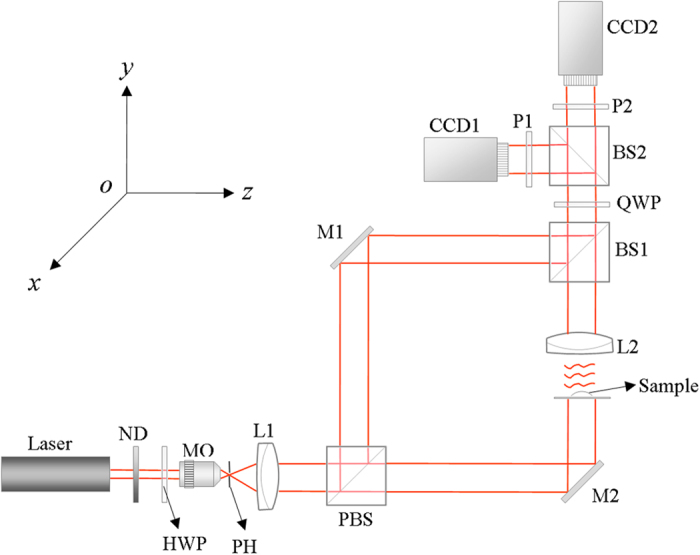
Configuration of the DCSPSI. ND, variable neutral density filter; HWP, half wave plate; MO, microscope objective; PH, pin hole; L1, L2, lens; PBS, polarized beam splitter; M1, M2, mirror; BS1, BS2, non-polarized beam splitter; QWP, quarter wave plate; P1, P2, polarizer.

Configuration of the DCSPSI system was illustrated in Fig. 1. It was based on a Twyman-Green interferometer adapted for transparent sample. A stabilized He-Ne laser was used as an illumination source. The variable neutral density filter (ND) can gradually change the intensity of laser. The half wave plate (HWP) was used to rotate the polarization direction of laser beam. The laser beam was divided into two orthogonal polarization beam by the polarized beam splitter (PBS) after spatial filtering, expanding and collimating in the usual manner as shown in Fig. 1, thus the modulation amplitude of interferogram on the CCD can be adjusted by PBS and HWP together. Then the transmitted beam (its polarization direction parallels to the *x* axis) which modulated the measured signal through the sample as the test beam, will pass through the imaging lens (L2) and be imaged on the two CCDs, and the reflected beam (its polarization direction parallels to the *y* axis) was used as the reference beam. The polarization interfering beams can be obtained when the test beam and reference beam arrive at the first non-polarized beam splitter (BS1). The quarter wave plate (QWP) with its fast axis at 45 degrees to the *y* axis was placed between the BS1 and the second non-polarized beam splitter (BS2). The polarization interfering beams will become orthogonal circular polarized beams after transmitting through the QWP. After that the second non-polarized beam splitter (BS2) was used to separate the polarization interfering beams into two parts. And then the two orthogonal interference fields were digitalized on CCD1 and CCD2 after through the first polarizer (P1) and the second polarizer (P2) respectively. There was a 45 degrees offset between the polarization direction of P1 and P2. Thus a pair of interferograms with the spatial phase shifts of π/2 can be obtained simultaneously by DCSPSI system at one-time single exposure.

DCSPSI can be easily applied in the measurement of a dynamic process. A pair of interferograms with the phase shifts of 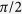 recorded by CCD1 and CCD2 can be respectively expressed as:


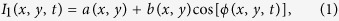



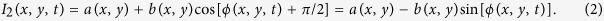


where 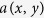 and 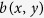 respectively denoted the background intensity and the modulation amplitude of interferogram, which can be considered as the unchanged parameters during the measuring process. 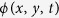 was the measured real-time phase, where *x* and *y* respectively represented the coordinates in CCD plane, and *t* denoted the time. By filtering out the background of interferogram with Gaussian high-pass filter^29^, Eq. (1) and Eq. (2) can be respectively rewritten as:


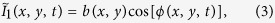



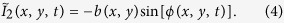


According to Eq. (3) and Eq. (4), the modulated real-time phase can be retrieved directly by an arctangent function as:


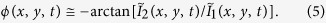


Note that the real-time phase included the phase of the measured sample and the additional phase 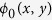 induced by the ambient medium and the DCSPSI system in Eq. (5). That is to say, to obtain the real phase of the measured sample, it was needed to eliminate the additional phase. To address this, we first captured a pair of interferograms without the sample and retrieved the additional phase. Usually, as long as the ambient medium was a homogeneous material, the real phase of the measured sample can be calculated by:


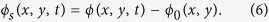


Thus the real-time height distribution of the measured sample can be expressed as^30^:


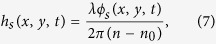


where 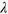 was the wavelength of the illumination laser, 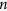 and 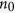 denoted the refractive indexes of the measured sample and its ambient medium, respectively. Assuming the sample was a homogeneous material, its volume can be calculated by


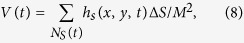


where 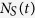 denoted the pixel number of the contact area of sample located on CCD at time *t*, 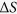 was the area of a single pixel of CCD, and *M* was the lateral magnification of imaging lens L2. After the real-time 3D shape of the measured sample was obtained, the corresponding 3D shape change will be determined easily.

## Results

In order to verify the performance of the proposed DCSPSI in a practical dynamic process, in this study, we implemented the evaporation process of a sessile droplet deposited on a glass slide in a natural condition. As shown in Fig. 1, a He-Ne laser with wavelength of 632.8 nm was used as the illumination source, the focal length of imaging lens L2 and the lateral magnification are respectively 120 mm and 2 × . Two identical CCDs with size of 576 (V) × 768 (H) pixels (576 mm × 768 mm) were employed to simultaneously capture a pair of interferograms. 4 sets of droplets of pure water (W1-W4) and 4 sets alcohol (A1-A4) with different initial mass, which were dropped on the glass slide by the pipette (Thermo Corp.), were used as the measured sample. The measurement was performed in an airtight laboratory with the temperature of 30 °C and the relative humidity of 60%, and the sampling interval about the evaporating pure water droplet or alcohol droplet was 50 ms, LabVIEW software(NI Corp.) was applied to control the data acquisition.

**Figure 2:**
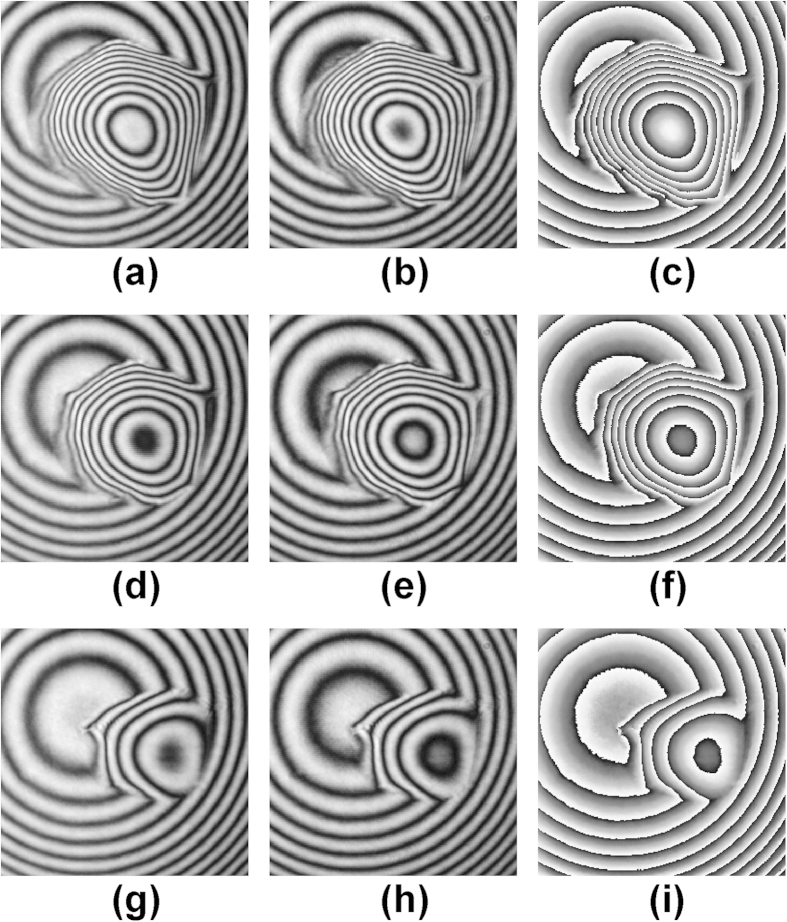
Interferograms (**a,b,d,e,g,h**) and the wrapped phase maps (**c,f,i**) of the water drop W1 at different time point: (**a–c**) at *t* = 0 s, (**d–f**) at *t* = 7.5 s, (**g–i**) at *t* = 15 s.

**Figure 3:**
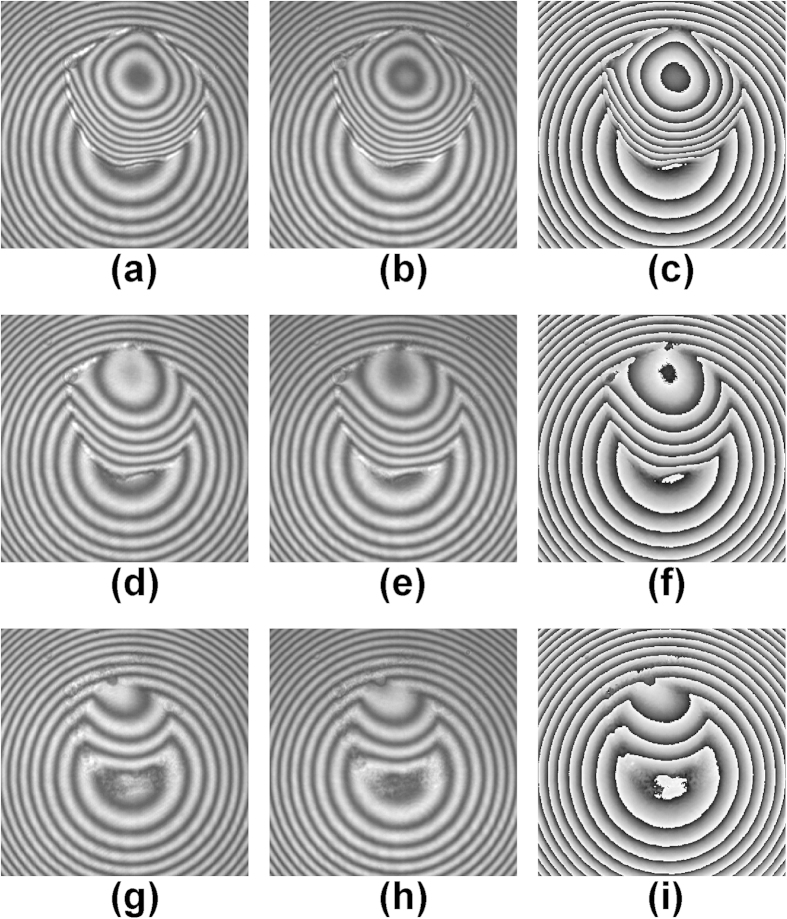
Interferograms (**a,b,d,e,g,h**) and the wrapped phase maps (**c,f,i**) of the alcohol drop A1 at different time point: (**a–c**) at *t* = 0 s, (**d–f**) at *t* = 7.5 s, (**g–i**) at *t* = 15 s.

Figure 2 showed a pair of interferograms with the phase shifts of π/2 and the wrapped phase retrieved by Eq. (5) of pure water W1, in which the size of interferograms was 256 × 256 pixels, and the time point of capturing a pair of interferograms by CCD1, CCD2 was at *t* = 0, 7.5, 15 s, respectively. Similarly, Fig. 3 also showed a pair of interferograms with the phase shifts of π/2 and wrapped phase retrieved by Eq. (5) of alcohol droplet A1, in which the size of interferograms was 361 × 361 pixels, and the time point of capturing a pair of interferograms by CCD1, CCD2 was at *t* = 0, 7.5, 15 s, respectively.

**Figure 4:**
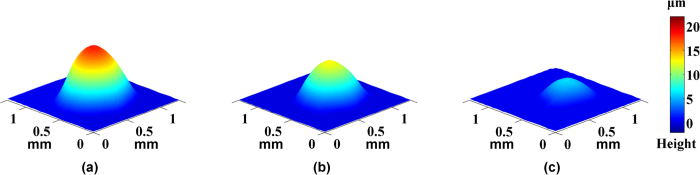
Height map of the water drop W1 at different time point: (**a**) *t* = 0 s, (**b**) *t* = 7.5 s, (**c**) *t* = 15 s.

**Figure 5:**
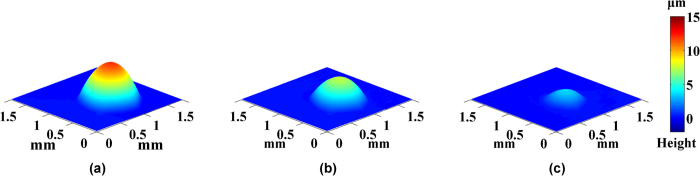
Height map of the alcohol drop A1 at different time point: (**a**) *t* = 0 s, (**b**) *t* = 7.5 s, (**c**) *t* = 15 s.

Using DCSPSI system, we first retrieved the additional phase 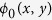, the real-time height and the volume of droplet by Eq. (6) and Eq. (7). Subsequently, Figs 4 and 5 respectively showed 3D shape of pure water droplet W1 and alcohol droplet A1. Multimedia Media1 and Media2 were the videos of evaporation process of pure water droplet W1 and alcohol droplet A1, respectively.

## Discussions

To understand the characteristics and the accuracy of the proposed DCSPSI method, as well as the law about the evaporation of a sessile droplet, we will analyze the evaporation process of 4 sets of pure water droplets and 4 sets of alcohol droplets with different initial mass. After the height distribution of the droplets were obtained, we will calculate the edge of the droplet with the numerical solution of the Otsu algorithm^31^, and the pixels number of the contact area of droplet. Thus the real-time volume of droplet can be calculated by Eq. (8). If the droplet was a homogeneous material, the real-time mass (named as transient mass (TM)) of droplet can be obtained by


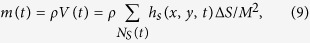


where 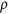 was the density of droplet, which was equal to 1 g/cm^3^ in pure water droplet and 0.8 g/cm^3^ in alcohol droplet.

**Figure 6:**
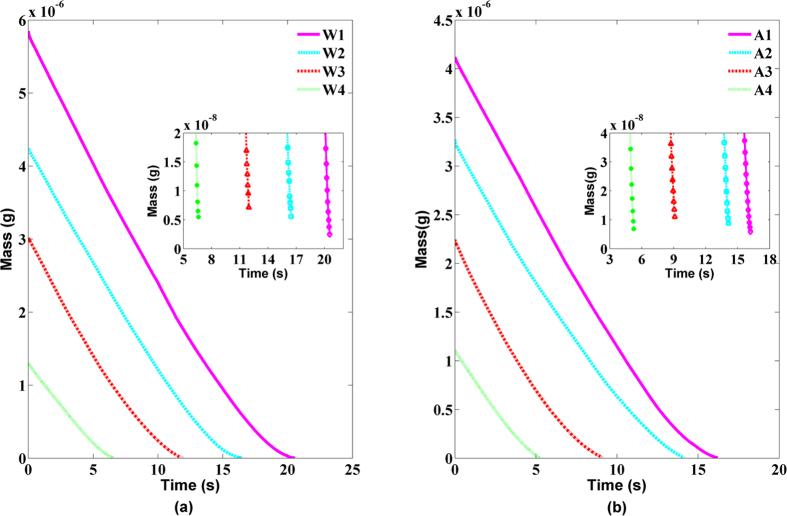
TM of the evaporating droplet changed with the time: (**a**) pure water, (**b**) alcohol (RH 60% and 30 °C).

**Figure 7:**
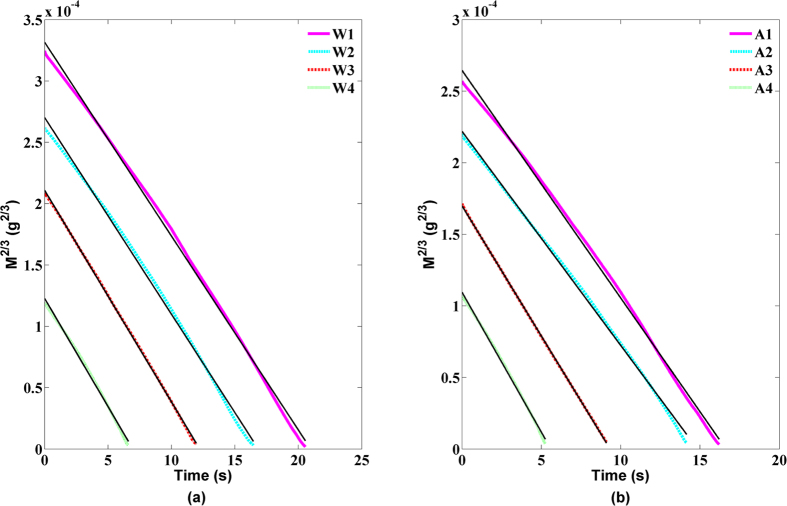
The 2/3 power of TM of the evaporating droplets changed with the time: (**a**) pure water, (**b**) alcohol, in which the black lines running through the data were the linear fit (RH 60% and 30 °C).

Figure 6(a,b) respectively showed the TM of the evaporation of 4 sets pure water droplets with different initial mass (W1-W4) and 4 sets of alcohol droplets with different initial mass (A1-A4), in which the small boxes denoted the data of evaporating droplet close to the end. We can see that the TM of the evaporating droplet still satisfied the 2/3 power law^32^,^33^ under a natural condition. Namely, the TM of droplet 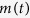, initial mass 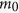 and the time 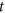 will satisfy the following expression:


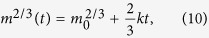


where 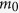 was the initial mass of droplet, *k* was a constant related to the diffusion coefficient of molecules in the air, the molecular weight of droplet, the contact radius, the contact angle of droplet, the universal gas constant, and the Kelvin temperature of environment. Using the above measured data of mass, we calculated the 2/3 power of mass 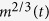 of the droplet evaporation. The obtained results are showed in Fig. 7, in which the color point presented the experimental data of 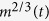 of the evaporating droplet, and the black line denoted the corresponding fitting data with the least square algorithm. The root mean square errors (RMSE) between the experimental data and the corresponding fitted data were shown in Table 1. We can see that all RMSE are less than 10^−6^ g^2/3^ or 10^−9^ g, indicating that our measuring result was consistent with the theoretical value coming from Eq. (10). Moreover, from Fig. 6 and 7, we can see that the smaller initial mass of the droplet is, the less difference between the experimental data and the theoretical value will be. For a large mass droplet, the experimental result will deviate from theoretical value. In the early stage of the droplet evaporation, its mass changed with time was linear, but in the late stage of the droplet evaporation, its mass changed with time will satisfy the 2/3 power law.

As we can see in the small box in Fig. 6, when the droplet evaporation reached the end of the droplet lifetime, for the evaporation process of droplet with microgram-level initial mass, even though in the case that the TM of droplet was less than 1‰ of initial mass, the obvious mass change (MC) of droplet can be measured, and the MC of droplet between two adjacent sampling interval can reach 10^−9^ g, and the corresponding volume change was 10^−12^ L. When the TM reached the end of the droplet lifetime, Table 2 presented the corresponding MC. We can see that the MC of a single pixel can reach 10^−12^ g, and the corresponding volume change was 10^−15^ L. These results demonstrated that the proposed method not only can perform the visual measurement for the entire process of the droplet evaporation, but also achieve the high measuring accuracy and sensitivity. If the lateral magnification of the DCSPSI imaging system was increased, the measuring accuracy and sensitivity of the proposed DCSPSI method will be improved.

**Figure 8:**
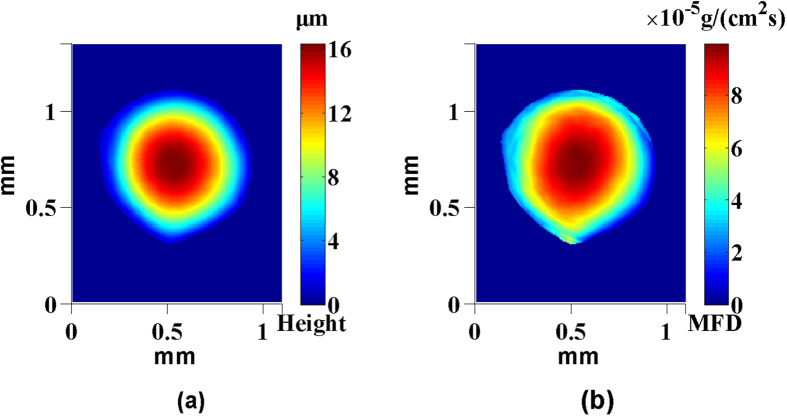
(**a**) Height map of the water droplet W2 at *t* = 0 s, (**b**) MFD on each pixel of the water droplet W2.

Following, we will analyze the mass flux density (MFD) of the droplet evaporation, in which MFD was defined as the mass change of droplet per unit area per unit time. From the above 3D shape data of droplet, we can obtain MFD on each region easily. The obtain results showed that the MFD of droplet was greatly related to the height of droplet. In addition, Fig. 8 showed the height distribution of W2 in early stage and its regional MFD, it was seen that the MFD of droplet were decreased as the height of droplet was decreased. Furthermore, we also presented the mean value of MFD of droplets in Table 3, it was observed that the mean value of MFD of droplet were related to the initial mass of droplet. That is to say, as the initial mass of droplet was decreased, the mean value of MFD was also increased.

Of course, the above measuring accuracy of DCSPSI system was determined under our current experimental condition. By improving the experimental condition and the performance of device such as reducing the interval of sampling time, choosing CCD with lower noise and the smaller pixel, using the imaging lens with larger magnification, or keeping the environment and the system more stability, thus the droplets with smaller mass also can be measured by the proposed DCSPSI system. Besides the convenience and high precision, DCSPSI also exhibited the great flexibility and versatility in measuring the evaporation process of droplet. Collectively, DCSPSI can not only measure the ideal droplet mode, but also the irregular contact surface and uneven evaporation of droplet. Moreover, since the analysis of the measuring data was very convenient, we can easily obtain important parameters to determine such as the contact surface shape, the global MFD, local MFD, contact angle of droplets, contact radius and so on.

## Conclusion

In this study, a novel measuring method of the droplet evaporation was proposed by DCSPSI system. The system employed polarization components and beam splitters to simultaneously generate two orthogonal interferograms with the phase shifts of π/2 on two CCDs, and then the measured phase can be real-time reconstituted by the two-step phase-shifting algorithm. The DCSPSI system preserved the advantages of visual, accurate, noncontact, nondestructive, and global in the optical interferometry, meanwhile offered the characteristics of fast, straightforward, and convenient. The measurement results of the droplet evaporation of pure water or alcohol with different initial mass showed that the DCSPSI can visually monitor the entire evaporation process of droplet. Under the condition of our laboratory, the measurable droplet volume can be from nanoliter to picoliter. The measuring precision and sensitivity can be greatly improved by increasing the magnification of the imaging lens, choosing CCD with faster acquisition speed and smaller pixel size. The obtained results showed that the 2/3 power of mass of non-idealized model droplets was linearly changed with the time, which demonstrated that the results satisfied the classical empirical formula. For the same droplets, the higher the height of droplet was, the greater the MFD will be; for different droplets in size, the larger the initial mass was, the smaller the MFD will be. Collectively, DCSPSI will provide a novel, simple and rapid method to study the evaporation process of droplet. DCSPSI not only can be used in symmetric ideal model droplets, but also can be applied to measure non-ideal model droplets under an open natural environment. In addition, this proposed DCSPSI method will supply a useful tool in monitoring cell culture, material surface tension and other dynamic measurements etc.

## Additional Information

**How to cite this article**: Sun, P. *et al.* Visual measurement of the evaporation process of a sessile droplet by dual-channel simultaneous phase-shifting interferometry. *Sci. Rep.* **5**, 12053; doi: 10.1038/srep12053 (2015).

## Figures and Tables

**Table 1:** RMSE between the experimental data and the fitted data of the evaporating droplets.

Droplets	W1	W2	W3	W4	A1	A2	A3	A4
*m*_*0*_( 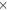 10^−6^ g)	5.85	4.25	3.02	1.30	4.02	3.20	2.19	1.08
RMSE( 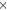 10^−6^ g^2/3^)	4.02	3.94	0.86	2.10	3.71	2.17	0.32	1.51
RMSE( 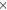 10^−9^ g)	8.06	7.83	0.79	3.03	7.15	3.19	0.18	1.86

**Table 2:** TM and MC for 8 sets of the evaporating droplets at the end of lifetime.

	t(s)	TM ( 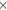 10^−9^ g)	MC ( 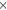 10^−9^ g)		t (s)	TM ( 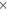 10^−9^ g)	MC ( 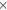 10^−9^ g)
W1	20.35	8.08	2.03	A1	16.00	13.17	2.98
	20.40	6.37	1.71		16.05	10.78	2.39
	20.45	4.92	1.45		16.10	8.85	1.93
	20.50	3.76	1.16		16.15	6.98	1.87
	20.55	2.47	1.29		16.20	5.89	1.09
W2	16.25	11.69	1.43	A2	13.95	19.72	4.11
	16.30	9.04	2.65		14.00	15.80	3.92
	16.35	7.96	1.08		14.05	12.99	2.81
	16.40	7.01	0.95		14.10	10.50	2.49
	16.45	5.57	1.44		14.15	8.79	1.71
W3	11.75	14.62	2.33	A3	8.90	23.74	3.98
	11.80	12.88	1.74		8.95	20.03	3.71
	11.85	10.96	1.92		9.00	16.27	3.76
	11.90	9.56	1.40		9.05	13.54	2.73
	11.95	7.14	2.42		9.10	10.99	2.55
W4	6.40	14.36	3.88	A4	5.05	19.67	5.30
	6.45	10.99	3.37		5.10	14.75	4.92
	6.50	8.11	2.88		5.15	10.54	4.21
	6.55	6.51	1.60		5.20	6.87	3.67
	6.60	5.49	1.02		5.25	3.72	3.15

**Table 3:** Mean MFD of the evaporating droplets with different initial mass.

Droplets	W1	W2	W3	W4	A1	A2	A3	A4
***m***_***0***_ ( 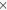 **10**^**−6**^** g)**	5.85	4.25	3.02	1.30	4.02	3.20	2.19	1.08
**MFD (** 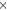 **10**^**−5**^ **g/(cm**^**2**^**s))**	7.73	7.65	8.78	12.26	5.64	7.51	10.15	12.90
